# Adaptive Immunity and Skin Wound Healing in Amphibian Adults

**DOI:** 10.1515/biol-2019-0047

**Published:** 2019-11-06

**Authors:** Antonella Franchini

**Affiliations:** 1 Department of Life Sciences, University of Modena and Reggio Emilia, Modena, via Campi 213/D, 41125 Modena, Italy

**Keywords:** skin wound healing, adaptive immunity, lymphoid organs, amphibians

## Abstract

Regeneration and repair with scarring of the skin are two different responses to tissue injury that proceed depending on the animal species. Several studies in multiple organisms have shown that the effectiveness of tissue repair gradually decreases with age in most vertebrates, while the molecular and cellular mechanisms underlying the diverse potentials remain incompletely understood. It is clear, however, that immune system actively participates in the whole process and immune-related activities can mediate both negative and positive roles to influence the quality and diversity of tissue response to damage. Compared with innate immunity, our understanding of the significance of adaptive immune cells in normal repair outcome is limited and deserves further investigation. Here, experimental evidence supporting the contribution of lymphocytes and the involvement of lymphoid organs in skin wound healing are discussed, focusing on the findings emerged in adult amphibians, key animal models for tissue repair and regeneration research.

## 1 Introduction

The healing of skin wounds involves a series of molecular and cellular events to restore the original structure and function. The ability to respond to injury and restore complex tissues varies widely among animal species, and in most vertebrates the capacity to regenerate and repair skin in a scar-free manner decreases at some time during ontogenesis [1, 2]. The causes of this decline are

not well understood. The comparative approach by using vertebrate models, has highlighted a main role of the immune system and the impact of inflammatory response in determining the outcome quality to tissue damage. Several immune cells and modulators are involved and influence the complex events of the healing process and, although it is believed that immunity correlates negatively with successful repair, there is evidence for positive effects in relation to the tissue, organ and developmental stage [3, 4, 5, 6, 7]. Another point of view suggests a correlation between development of a functional immune system and a gradual loss of regenerative capacity [3, 8]. A supporting example is provided by amphibian adults: urodeles (*i.e*. newts, axolotls), that retain regeneration-competence throughout life and anurans (*i.e. Xenopus*), whose regenerative ability is restricted to larval stages, have immune systems with different complexity. By comparison, urodeles are relatively “immunodeficient” with poorly efficient adaptive responses, while *Xenopus* matures a sophisticated adaptive immunity whose components are similar to those of mammals [3, 6, 9]. The repair process of the skin after wounding consists of several phases and involves dynamic communications between resident and migratory cells and the extracellular matrix components. The immune cell types, mobilized in the early inflammatory response, change spatially and temporally and the lymphocytes, especially T cells, are the last cells recruited to an injury site [10, 11, 12]. The exact contribution of types and subsets of adaptive immune cells remains unclear and studies concerning the participation of lymphoid tissues have received little attention. Here, the healing of skin wounds in amphibian adults and the involvement of lymphoid organs (such as the thymus and spleen) in the anuran *Xenopus laevis*, a key model organism for tissue repair/regenerative and comparative immunological research [9], are considered. Adaptive immune cell response in adult tissue repair is also discussed.

## 2 Skin wound repair in adult amphibians

Studies in *X. laevis* demonstrated that young froglets (after metamorphosis) were able to regenerate skin wounds without fibrosis and scar formation, just like axolots and mammalian embryos where the healing was associated with almost no acute inflammation and immune cell infiltration [13, 14, 15, 16]. The contribution of early markers for limb blastema cells, such as the expression of paired homeobox transcription factor *prx1* and activation of *prx1* limb-specific enhancer, was demonstrated in froglet scarless healing which has been proposed to proceed with mechanisms common to initial limb regeneration (14). In salamanders, an effector bioactive peptide that may directly promote the quick skin wound healing has been identified [17]. It accelerates re-epithelialization and granulation tissue formation in the injury site, by increasing motility and proliferation of several cell types (keratinocytes, vascular endothelial cells, fibroblasts), and promotes the release of cytokines. It also quickens the healing of full-thickness wounds in mice [17]. It should be underlined that the skin of several amphibians is rich in dermal granular glands producing antimicrobial peptides that play a crucial role in the repair process [18]. The infection is a main factor that hinders the repair; organisms have developed many host defense molecules to control microbial proliferation and immune response to biological or physical insults. Skin defenses have been studied to clarify the immune responses to environmental pathogens, i.e. chytrid fungi, that infect amphibians thus contributing to their global population declines. In addition to bioactive peptides, other components of constitutive defences include enzymes, immunoglobulins and antifungal metabolites produced by symbiotic skin bacteria [19].

The regenerative potential of young froglets decreased during the anuran growth: in 15 month old *X. laevis*, the organization of a granulation tissue, involvement of myofibroblasts, formation of a fibrotic dermis and scar-like tissue were observed [12]. Similarly, in adult *Rana catesbeiana* the repair proceeded with wound contraction and scar tissue and the transition from skin regeneration (observed in tadpoles) to scar synthesis was proposed to occur after the initiation of metamorphosis [20]. Imperfect structure of the stratum compactum, not equivalent to that of unwounded dermis, was also found in other adult frog species [21]. Conversely, in addition to a very weak immune response in the wound area, the scar-forming fibroblasts expressing α-smooth muscle actin, the myofibroblasts, were absent during the rapid and perfect skin healing in axolotls [13]. In *X. laevis*, the post-wounding inflammatory response persisted over time, was resolved with delay, and involved several immune cells that were immunoreactive for versatile immune players, *i.e*. tumor necrosis factor (TNF)-α, transforming growth factor (TGF)-β, inducible nitric oxide synthase (iNOS). By quantitative PCR analysis, the expression patterns of immune genes, *i.e. Xenopus suppressor of cytokine signaling* (*XSOCS)-3, TGF-*β*2* (*XTGF-*β*)2*, were particularly up-regulated when many inflammatory cells were present in the granulation tissue [12]. SOCS proteins were recognised as cytokine-inducible negative feedback inhibitors and shown to act as physiological regulators of inflammation and adaptive immunity [22]. Elevated levels of SOCS-3 mRNA were also demonstrated in mammal models of impaired skin healing [23] and continued gene expression was detected at limb regeneration-incomplete, compared to the regeneration-complete, stages of *Xenopus* larvae [24]. Different actions have been characterized for each isoform of TGF-β, the main pleiotropic mediator required in repair process. In particular, TGF-β1 and -β2 were found to be essential for collagen and other extracellular matrix component deposition and organization and to induce the fibroblasts to myofibroblast transition and fibrotic scarring response. High levels of these factors were reported during scar-forming repair in adult mammals, while the low expressions observed in mammalian embryos and axolotls were associated with a scar-free healing [13, 25, 26].

## 3 The thymus and skin wound repair

Skin repair in *X. laevis* adults (15 month old) has been associated with morpho-functional modifications of the thymus and the changes were particularly evident when the lymphocytes, most of which positive to specific T cell markers, were found in the wound granulation tissue [27]. The organ significantly increased in size 14 day post injury; dilated blood vessels, areas with densely packed thymocytes and corpuscles (not observed in control thymus) similar to mammalian Hassall’s bodies were detected in medulla. More numerous mucocyte-like cells, epithelial cysts, clusters of myoid cells and cells immunoreactive to anti-TNF-α (cortico-medullary dendritic, medullary epithelial, granular basophilic and myoid cells) were also observed. The response of the microenvironmental cells, known to be involved in modulation of thymocyte differentiation, allows to suggest a stimulation of thymus activity that could be related to T lymphocyte infiltration into wound connective tissue [27]. In contrast, the histological patterns observed in the thymus from tadpoles undergoing metamorphosis, whose potential to regenerate a correctly patterned tail declined, were correlated to a reduced organ functionality [28, 29]. It is likely that at different stages of *Xenopus* development distinct molecular and cellular mechanisms may be involved in the progressive loss of regenerative ability.

## 4 The spleen and skin wound repair

**Figure 1:**
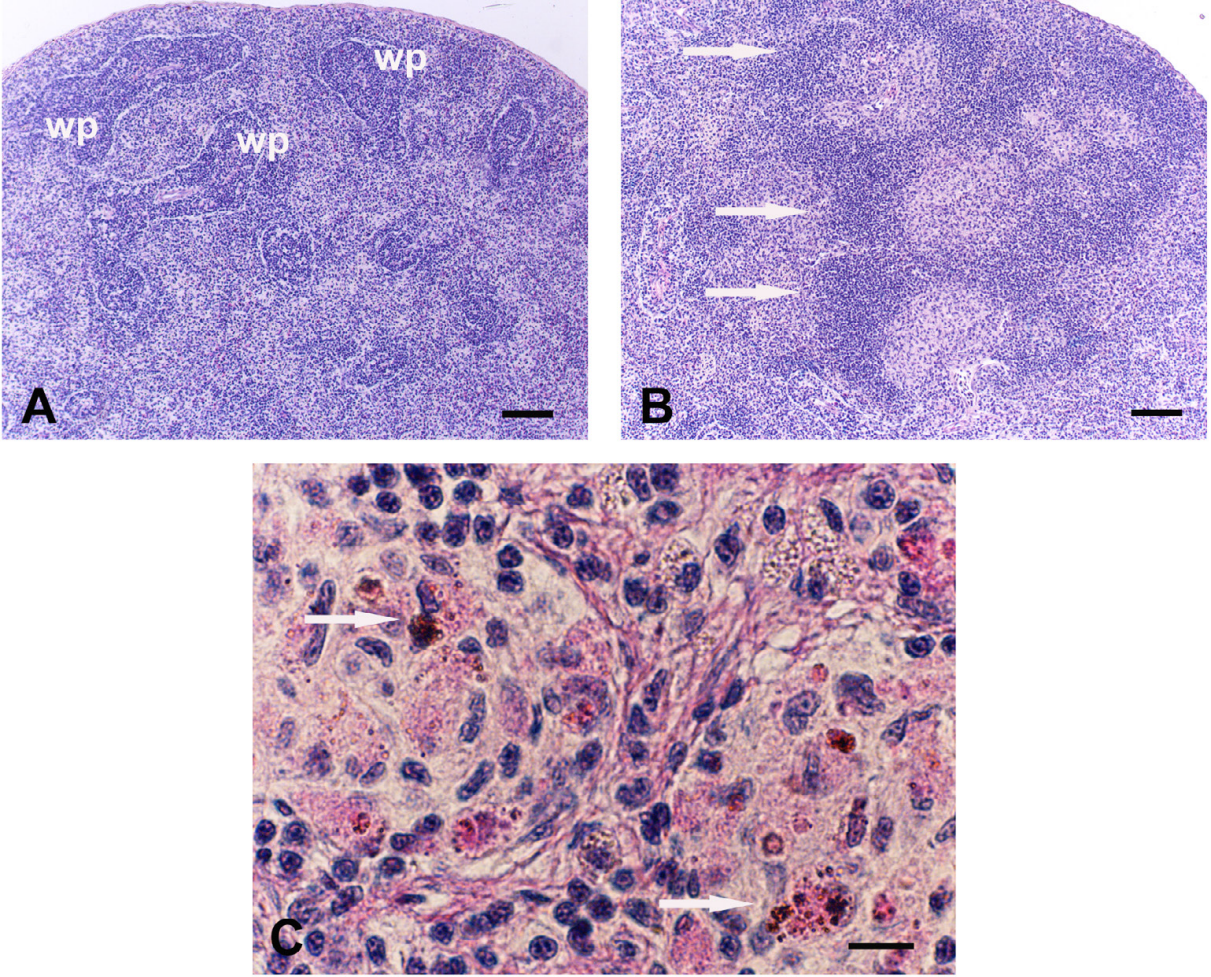
Light micrographs of the spleen of 15 month old *X. laevis* adults. The comparison between the sections from unwounded (**A**) and operated (14 day after the skin wounding) frogs (**B**) shows structural changes in the white pulp (wp): note the well developed lymphoid follicles with central light cores (arrows in **B**). **C)** The melanomacrophage centers (arrows), induced during the repair process, are indicated in splenic white pulp. The sections were stained with PAS/hematoxylin reaction, scale bars = 100 μm (**A**, **B**) and 10 μm (**C**).

The morpho-chemical responses of the spleen during cutaneous wound healing in 15 month *X. laevis* were also studied to explore the role of mature adaptive immunity [30]. Gradual and transient structural changes were detected, especially 14 days post-injury when the splenic size significantly increased. Of note, in white pulp the developed perivascular sheaths were associated to lymphoid nodules with central light areas (compare Figure 1A and 1B) structurally similar to the mammalian germinal centers. In the core regions of these active follicles, mitotic cells, apoptotic cells, isolated large immature cells and pigment-containing cells were found. Moreover, a higher number of stromal cells immunoreactive to anti-cytokines (TNF-α, TGF-β1) and -iNOS antibodies, induced from the first days post-injury, were organized in a network of non-lymphoid cells the 14^th^ day. The positive leukocytes (*i.e*. basophils, neutrophils and macrophages) in red pulp and lymphoid cells reactive to T cell specific antibodies within the perifollicular zone were also found more frequently. During the repair process, the pigment cells, called melanomacrophages in poikilotherm lymphoid tissues [31], were observed packed in organized melanomacrophage centers (MMC) (Figure 1C). The structure and immunohistochemical patterns gradually restored to those of controls [30].

## 5 The adaptive immune response in tissue repair

The post-injury activation of adaptive immune system follows the innate response and the lymphocytes were the last cells to infiltrate the wound site [10, 11, 12]. In *Xenopus* adults, the thymus activity seemed to be stimulated and T-lymphocytes were detected during tissue maturation

phase of skin repair. The thymic response mainly involved medullary microenvironmental cells, such as the muscle-like myoid cells, an increased TNF-α immunoreactivity and, interestingly, the induction of Hassall’s body-like structures [27]. In mammals, Hassall’s corpuscles were suggested to actively participate in thymic physiological activities, mature developing thymocytes and act in instructing dendritic cells, by secretion of lymphopoietin, to differentiate regulatory T cells (Tregs) [32, 33]. The myoid cells produce lympho-proliferative cytokines, protect thymocytes from apoptosis, by activation of pro-survival signaling pathways, and modulate their differentiation [34, 35]. There is also evidence that, despite the complexity of the molecular factors involved, several TNF superfamily signals, such as TNF-α, and TNF receptor family signaling, are required to shape, maintain and control the functions of thymic stromal microenvironment [36, 37]. Moreover, this cytokine, constitutively produced in thymus, play a main role in regulation of thymocyte production and development of thymus-derived Tregs [38, 39].

Regarding the contribution of T cells in wound healing, studies in mammals support a critical modulatory function by the secretion of distinct lymphokines and direct interactions with resident and non-resident cells at the injury site [11, 40, 41]. In T-cell depleted mice, the healing resulted impaired with reduced mechanical strength and collagen deposition [42] and in adult athymic nude mice, the skin repaired without scarring and the external ears regenerated by forming a blastema [16, 43]. Despite these results, studies in different immunodeficient mice have suggested that the absence of thymus and/or T cells may not fully explain the regenerative ability in these animals [44]. Experiments of specific cell depletion demonstrated that T cell subsets influenced the repair outcome with different effects, *i.e*. up-regulatory for CD4- and down-regulatory for CD8-lymphocytes [40, 45]. Both subtypes, however, did not seem to play a critical function: deficiency of CD4-or CD8-cells modified the infiltration of inflammatory cells and cytokine expression levels but did not impair skin healing [46]. The immunoregulatory contribution of tissue-resident T lymphocytes, the γδT cells, through local production of growth factors and cytokines, is now also appreciated [47, 48]. A role in promoting tissue repair and regeneration is emerging for a specialized sub-population, the Tregs [49]. In *Xenopus* tadpoles, the recovery of tail regenerative potential, after its loss in the refractory period, coincided with the transient infiltration of *foxp*3-expressing Tregs in the amputated stumps to suppress the function of immune cells that impaired the repair ability [50]. In mammals, Tregs were suggested to facilitate wound healing in several injured tissues, limit pro-inflammatory responses and promote the wound closure in skin repair by using the epidermal growth factor receptor pathway [49, 51, 52]. The involvement of adaptive immune responses in regenerative process of urodeles, which are considered to lack some advanced functions found in adaptive immunity of adult frogs [6], has not yet been clarified. In salamanders, the inhibition of limb regeneration by the immunosuppressive drug, Cyclosporin, can be recovered with Interleukin 2, a key cytokine regulating T lymphocyte differentiation, thus suggesting a role for effectors of cellular immunity [53].

Little is known about the B-lymphocytes detected within injured adult tissue [54, 55]. B cells can work as modulators of tissue repair in view of the multiple functions. In addition to the capability to differentiate into antibody-secreting cells, B cells can present antigen to T cells and up- or down-regulate local immune responses through production of pro- or anti-inflammatory cytokines [56]. Studies performed in splenectomized mice revealed positive regulative effects of these cells and the necessity of the spleen in cutaneous wound healing. In these models, the healing process was prolonged, antibody binding to damaged tissues reduced and the repair ability restored by transfer of spleen cells [57]. The B-cells were proposed to promote the healing process by their stimulation to produce cytokines and growth factors through Toll-like receptor-4 signaling in a CD19-dependent manner [58]. Recently, it has been shown that topical application of mature naïve B cells to the wound bed was able to modify the injury microenvironment to improve the quality of skin repair [59]. The involvement of B1-cells, a minor fraction of splenic B-lymphocytes, was also reported: these cells infiltrated into the wound and downregulated the inflammatory response by the production of interleukin-10 [60]. The studies in *Xenopus* adults further support the role of the spleen, that represents the main secondary lymphoid organ of this species [9]. Indeed, during the repair process, the spleen underwent structural and molecular modifications that may be related to the concomitant stimulation of thymus activity [27, 30]. Of interest, transient structures similar to germinal centers were induced within the lymphoid follicles, MMC were organized and high numbers of anti-CD3ε positive lymphocytes were present in the perifollicular regions. The development of splenic T- and B-dependent compartments and induction of molecules, *i.e*., cytokines and iNOS, could help the interactions between components of the humoral immune responses [30]. It should be underlined that splenic white pulp has become more complex in terms of microarchitecture, cellularity and functional capacity over the course of vertebrate evolution, and it is believed that the germinal centers are not present until the appearance of birds [61]. However, although *Xenopus* B cell response seems to occur in the absence of defined germinal centers, antigen dependent B cell maturation involves somatic hypermutation and class switch recombination mediated by the activation-induced cytidine deaminase, enzyme that was detected in follicular B cell areas [62]. Interestingly, preliminarity data on histological similarities and functions have led to the hypothesis that the MMC, found primarily in poikilotherm lymphoid tissues, could be primitive evolutionary precursors of mammalian germinal centers, and may be considered histological indicators of an immune response [31]. In addition, only a single subset of conventional dendritic cells, named XL cells, has been described (based on morphology and surface phenotype) in frog splenic white pulp. These cells were observed to carry out activities of both conventional and follicular dendritic cells as the prototype for antigen presentation to B cells. They acquire and present native antigens to B cells, interact with T cell then with B cells (after white pulp immigration) and produce B cell chemoattractant and pro-survival factors [63, 64, 65].

## 6 Conclusion

In summary, there is a growing body of evidence to support the idea that local immunity and inflammation strongly influence the quality of wound repair, as well as an associated contribution of adaptive immune cells. The studies in *Xenopus* adults have indicated the involvement of primary and secondary lymphoid organs in response to tissue injury. Moreover, it should be emphasized that the development of a functional immune system can be correlated with the progressive decline of scar-free healing ability. In adult life, anurans mature a more sophisticated adaptive immunity than the highly regenerative urodeles which are thought to have a poor adaptive immune efficiency [3, 8, 9, 66]. Further investigations of cellular and molecular differences and similarities of repair processes in diverse animal models, could help to understand how to prevent and control scar tissue formation and promote regenerative responses in human adults.
